# Advances in intranasal application of stem cells in the treatment of central nervous system diseases

**DOI:** 10.1186/s13287-021-02274-0

**Published:** 2021-03-24

**Authors:** Yu-Ting Zhang, Kai-Jie He, Jin-Bao Zhang, Quan-Hong Ma, Fen Wang, Chun-Feng Liu

**Affiliations:** 1 Department of Neurology, The Second Affiliated Hospital of Soochow University, Suzhou, 215004 China; 2 Institute of Neuroscience, Soochow University, Suzhou, 215123 China

**Keywords:** Intranasal application, Central nervous system, Stem cells

## Abstract

Stem cells are characterized by their self-renewal and multipotency and have great potential in the therapy of various disorders. However, the blood–brain barrier (BBB) limits the application of stem cells in the therapy of neurological disorders, especially in a noninvasive way. It has been shown that small molecular substances, macromolecular proteins, and even stem cells can bypass the BBB and reach the brain parenchyma following intranasal administration. Here, we review the possible brain-entry routes of transnasal treatment, the cell types, and diseases involved in intranasal stem cell therapy, and discuss its advantages and disadvantages in the treatment of central nervous system diseases, to provide a reference for the application of intranasal stem cell therapy.

## Background

Central nervous system (CNS) diseases, such as cerebrovascular diseases, Alzheimer’s disease (AD), and Parkinson’s disease (PD), mainly involve the injury, degeneration, and loss of neural cells. Stem cells are a type of cells with the potential of self-renewal and multipotency, which exhibit great potential in replacing damaged neural cells and improving neurological function [1]. Stem cell therapy has great clinical prospects in the treatment of CNS diseases, with several ongoing clinical trials [2].

The blood-brain barrier (BBB) is an obstacle for delivery of most drugs to the CNS via traditional treatment routes (such as intravenous injection) that reduces therapeutic efficacy of most drugs. Development of compounds that can bypass the BBB is always a challenge for drug development. Stem cell transplantation has therapeutic potential in various diseases such as PD and AD. However, stem cells therapy in the brain always requires an invasive approach, such as stereotactic or intrathecal injection. The invasive approach limits the application of stem cell transplantation in the therapy of neurological disorders. Many studies have observed that intranasal application (INA) can bypass the BBB and enable the delivery of small molecular substances and macromolecular proteins to the CNS [3, 4]. Moreover, compared with stereotactic brain injection and intrathecal injection, INA is less invasive and can be done repeatedly. Intranasal delivery of drugs to the brain was first established by William Frey in 1989 [5]. Since then, a lot of other substances such as chemicals [6], plasmids [7], peptides [8, 9], viruses [10], and metals [11] have been delivered successfully through the intranasal cavity. In 2009, Danielyan et al. firstly delivered fluorescently labeled rat mesenchymal stem cells (MSCs) or human glioma cells to rodent brains via the nasal cavity. The intranasally delivered cells were observed in the olfactory bulb, cortex, cerebellum, and subarachnoid space [5]. Since then, several studies have shown that stem cells derived from different tissues can also be delivered to the CNS by INA, exhibiting therapeutic effects [12–15]. The intranasally delivered cells can be observed in the olfactory bulb, cortex, cerebellum, and subarachnoid space [12]. The intranasally delivered cells even exhibit more extensive migration to the injured or damaged areas in murine models of hypoxia-ischemia and ischemic stroke [16, 17], or brain tumors [18, 19]. In contrast, they seem not to localize to the subventricular zone; the region where endogenous adult stem cells are enriched [17]. However, it is worth noting that intranasally delivered stem cells show a low migration efficiency since a large number of cells remained in the upper nasal cavity 1 h after intranasal administration [12, 14]. Here, we review the possible brain-entry routes and the types of stem cells in the intranasal treatment of CNS diseases. Due to limited space, our current review only discusses INA of stem cells, rather than other substances that can be delivered via INA, which are reviewed elsewhere [20–25]**.**

## Possible brain-entry routes of INA

**Fig. 1:**
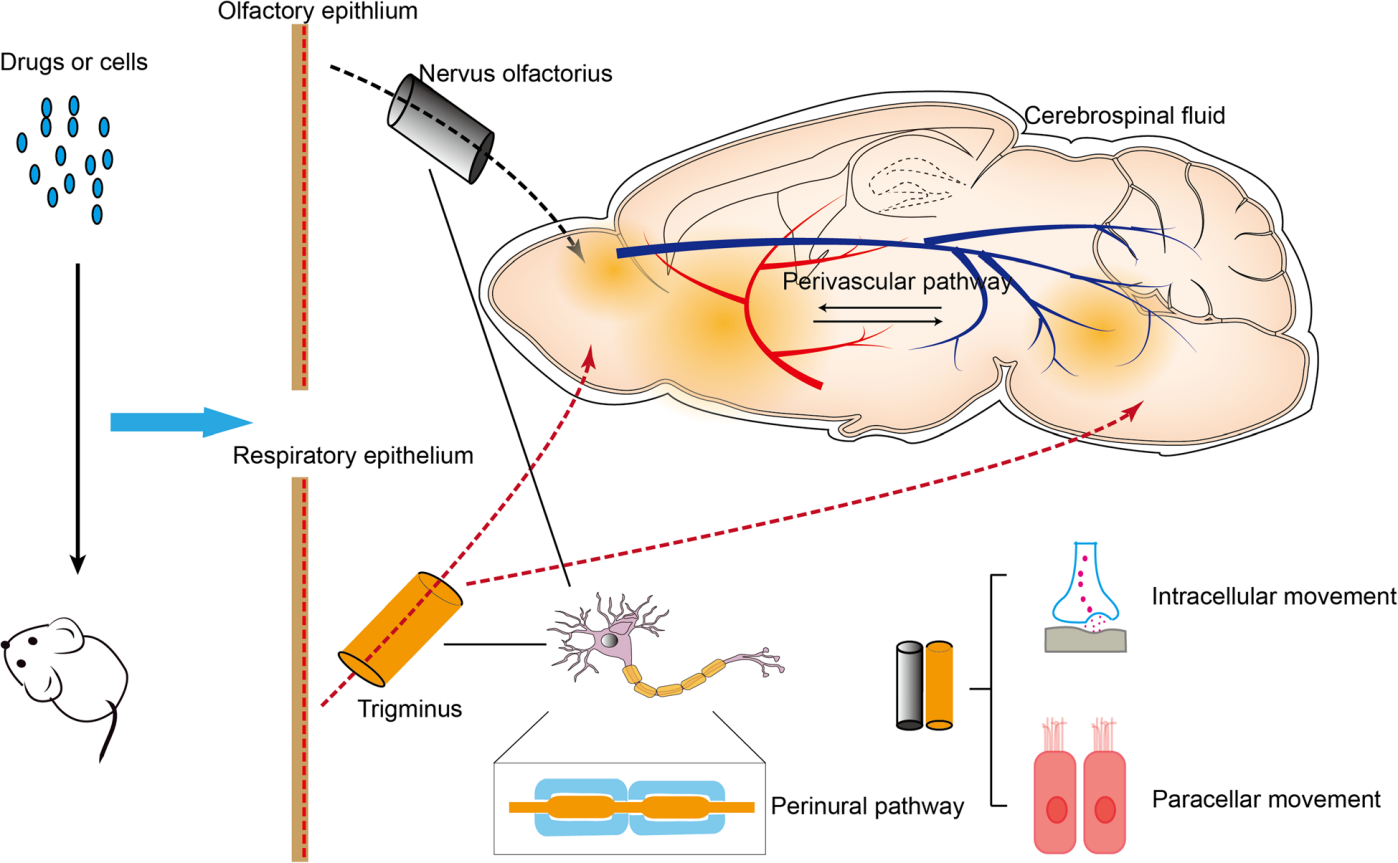
Possible brain-entry routes of substances by INA (in rodents as an example). After intranasal injection into the nasal cavity of rodents, drugs or cells are transported via the intracellular and/or paracellular pathways, and pass through the olfactory epithelium and reach the olfactory bulb along the olfactory nerve, or pass through the respiratory epithelium to reach the olfactory bulb/brainstem along the ophthalmic or maxillary branches of the trigeminal nerve. After reaching the CNS, drugs or cells are further distributed in the brain through the cerebrospinal fluid and/or perivascular spaces

Since 1989, Frey proved the existence of the route from the nose to the brain and opened a new era for INA [5]. In INA, substances deposit in the epithelial cell layer of the nasal cavity and reach the brain parenchyma either directly by migrating along the olfactory or trigeminal nerve, or through the cerebrospinal fluid circulation [24]. Once the substances reach their specific location, they can be transported to the CNS through the intracellular and paracellular transport pathways (Fig. 1).

### Intracellular transport pathway

Receptor-mediated endocytosis is the main mechanism by which substances enter the CNS through the intracellular transport pathway. Thorne et al. found that horseradish- peroxidase-conjugated wheat germ agglutinin (WGA-HRP) could reach the brain parenchyma via receptor-mediated endocytosis of the olfactory receptor neurons (ORNs) [26]. After being internalized by ORNs, the substances form vesicles and move along the axons. Once the vesicles reach the nerve endings, the substances are released by exocytosis to reach the periglomerular cell layer of the olfactory bulb. There are also endocytosis-mediating receptors on the surface of mitral cells within the periglomerular cell layer, which produce secondary transport of the substances. Through the above process, substances can diffuse to multiple brain regions, mainly including the olfactory nodule, piriform plexus, amygdala, hypothalamus, and inner olfactory plexus [27]. In addition to the ORNs, Robertson et al. [28] and Anton et al. [29] confirmed that WGA-HRP enters the CNS through neuroendocytosis of the ophthalmic and maxillary branches of the trigeminal nerve.

Distribution of substances in the CNS can be observed within a few hours after intranasal injection. In contrast, it takes longer for substances to enter the CNS via the intracellular transport pathway. In addition, the characteristics, molecular weight of the substances, and the selectivity of receptors limit the transportation efficiency [21], suggesting that intracellular transport may not be the main route after INA. Further investigations are required to validate whether stem cells enter the brain through the intracellular transport pathway.

### Paracellular transport pathway

In physiological structures, cells connect with one another through different structures, such as the common tight junctions [30]. These connections are innately impermeable to macromolecular drugs. However, in the nasal cavity, they are permeable due to the continuous renewal of the neurons and basal cells, which provides the basis for paracellular transport. Through the paracellular transport pathway, drugs can reach the olfactory bulb and other areas of the CNS within a few to 30 min [31].

#### Perineural pathway

In the nervous system, oligodendrocytes in the CNS and Schwann cells in the peripheral nervous system form myelin sheaths, which surround the axons or the long dendrites of sensory neurons. The space between the myelin sheaths and neurons is known as the perineuronal space. Myelin sheaths have plasticity and play an important role in nerve signal transduction. Additionally, they support and guide neuron migration and participate in substance metabolism and nutritive function [32]. They also form the physiological basis for substances to enter the CNS from the olfactory epithelium along the olfactory nerve or from the respiratory epithelium along the trigeminal nerve. The paracellular pathway of drugs delivered intranasally starts from the paracellular fissure of olfactory epithelial cells (Fig. 1). The tight junctions between the olfactory neurons and the supporting cells enable substances to move from the nasal cavity to the lamina propria [33]. Most of the substances are absorbed from the lamina propria by local blood vessels or lymphatic vessels and enter the cervical lymph nodes. The remaining substances are transported through the perineural space, along the olfactory nerve, and terminate at the olfactory bulb. Substances can also move along the trigeminal nerve, pass through the cribriform plate, and reach the olfactory bulb or brain stem [34]. A previous study showed that MSCs could pass through the cribriform plate and be detected in the olfactory nerve layer of the olfactory bulb 2 h after nasal administration [14], but the mechanism remains unclear.

#### Perivascular pathway

Perivascular spaces play an important role in the transport of substances to deep parts of the brain after arriving in the CNS via the perineural pathway, especially in the process of rapid distribution of small molecules in brain parenchyma at the early stage [35]. Perivascular spaces are formed between the endothelium of large and medium-sized arteries and veins in the brain and the glial cells surrounding them. In research on the brain lymphatic system, Iliff et al. suggested that most cerebrospinal fluid from the subarachnoid space flows back to brain parenchyma through perivascular spaces and exchanges with brain tissue fluid [36]. A similar distribution was observed between tracers injected intranasally and via the subarachnoid space, suggesting that perivascular spaces participate in the transport route after INA [37, 38]. This raises the question whether stem cells could distribute in the brain through this pathway. Although the structure of perivascular spaces suggests that it is difficult for macromolecular substances to exist in perivascular spaces [31], some results have shown that colocalization of MSC markers and the vascular endothelial marker von Willebrand factor is observed 7 days after injection of MSCs [14]. Endogenous stem cells can migrate from the subventricular zone (SVZ) to the olfactory bulb through rostral migration using perivascular spaces as a scaffold [39]. Whether INA of exogenous stem cells can take advantage of this pathway is a question worthy of further investigation.

## Types of stem cells administered by transnasal route

No study has compared the function of all types of stem cells at the same time. Some in vivo investigations have shown that the fusogenic role of microglia could be even more important after neural stem cell transplantation into brains affected by neurodegenerative diseases associated with microglial activation. Among cultured stem cells in vitro, it has been clearly shown that embryonic stem cells readily fuse with microglia. These microglia then, in turn, go on to fuse with mature neurons [20]. However, definitively demonstrating which type of intranasally delivered stem cells perform best in reaching appropriate targets and have the best restorative or therapeutic effect may be difficult. We agree with the conclusion of Danielyan et al. that the motility and migratory potential of stem cells can serve as predictors of their therapeutic efficacy because they all relate to the functional heterogeneity of stem cells [40].

### Neural stem cells (NSCs)

NSCs have the ability to differentiate into neurons and glia, thus harboring advantages in the treatment of neurological diseases. NSCs can be obtained from either isolating the cells from the SVZ of newborn or E14 rats [41] or from differentiation of other pluripotent stem cells such as hematopoietic stem cells, embryonic stem cells, MSCs, and induced pluripotent stem cells (iPSCs) [42]. INA of NSCs has been used for treatment of a variety of neurological diseases. For example, INA of NSCs, combined with other therapy, increases the survival in a murine model of glioblastoma of the brain (GBM) [19, 43, 44]. Intranasal injection of NSCs has also been used in the treatment of brain injury [45] and PD [46]. However, in terms of clinical application, as the main source of human NSCs is fetal brain tissue, its clinical application is limited by ethical issues [13].

### MSCs

MSCs are adult stem cells derived from various sources including the bone marrow, fat, umbilical cord, and cord blood. Bone marrow-derived MSCs (BM-MSCs) contain a subgroup expressing nestin, a marker of NSCs, and can differentiate into glial cells and neurons with complete functions, promoting nerve tissue regeneration [47]. Therefore, INA of BM-MSCs has been widely used in research into treating brain injury [48], neurodegenerative diseases, and glioma [15]. Several clinical trials are ongoing related to therapy of CNS diseases by intranasal injection of MSCs.

In addition to BM-MSCs, some recent studies have explored the therapeutic potential of intranasal injection of dental pulp stem cells (DPSCs) in treating PD [49]. DPSCs are neural-crest-derived ectodermal MSCs with strong self-renewal and multipotent capacity. They can be isolated from extracted dental pulp easily and show great potential in the treatment of CNS diseases and retinopathy. Endometrial MSCs are also used in PD therapy [50].

### iPSCs

iPSCs are a special type of stem cells that are engineered from somatic cells, and their properties resemble inner mass cells of the embryo. iPSCs are generated from the somatic cells through genetic engineering, by which somatic cells are reprogrammed to cells with pluripotent capacity similar to that of embryonic stem cells. This technology was first introduced by the Japanese scientist Yamanaka in 2006 [51]. The induction of NSCs or other nerve cells from autologous iPSCs reduces the immunogenicity of iPSCs, thus immune rejection after transplantation, and resolves the ethical issues of transplanting NSCs. Many studies have shown that INA of iPSCs can inhibit acute or chronic airway inflammation [52–54], but studies of INA of iPSCs or iPSC-derived cells in neurological diseases is still lacking.

## INA of stem cells for therapy of CNS disorders

No study has shown that there are particular diseases that would benefit most from INA compared with other administration routes. Obviously, it is difficult to compare all diseases and all administration routes in the same study. Although INA it has no disease specificity, it also shows the advantages of its wide application in a variety of neurological diseases. INA offers advantages over intravenous or other routes of administration in several circumstances [13]: (1) it has a higher degree of delivery to the CNS; (2) it avoids first-pass metabolism; (3) it is noninvasive and easy to be administered, which allows repetitive administration if necessary; and (4) its adverse effects can be minimized since other healthy organs are not exposed to the therapeutic compound. These properties make intravenous administration one of the prime strategies for therapy of long-term neurological disorders with multiple pathological regions, such as AD and PD.

Neuroinflammation is an important factor in the occurrence and development of neurological diseases, especially in PD and AD. However, only drug treatment for neuroinflammation in neurological diseases is very hard to achieve the best. Fortunately, an increasing number of studies have confirmed that stem cells transplanted into the brain can regulate the microenvironment of lesions and alter inflammatory mediators, so as to reduce the symptoms of neurological diseases. Some studies have suggested that INA of stem cells can also play an anti-inflammatory role [55]. For example, Danielyan L et al. [56] reported noninvasive INA of MSCs to the brains of unilaterally 6-hydroxydopamine (6-OHDA)-lesioned rats. After 40–110 days of INA of stem cells, MSCs decreased the concentration of inflammatory cytokines interleukin (IL)-1β, IL-2, IL-6, IL-12, tumor necrosis factor, interferon-γ, and granulocyte–macrophage colony-stimulating factor in the lesioned side to their levels in the intact hemisphere. McDonald et al. [57] found that INA of a clinically relevant dose of human umbilical-cord-derived MSCs in a rat model of neonatal hypoxic–ischemic brain injury was neuroprotective by restoring neuronal cell numbers and reducing brain inflammation.

### Neurodegenerative diseases

The common neurodegenerative diseases include PD, AD, Huntington’s disease, and amyotrophic lateral sclerosis, which are characterized by neuron degeneration and loss in the brain and/or spinal cord. In the treatment of neurodegenerative diseases, stem cells can replace degenerative cells or protecting cells via transferring neurotrophic factors [58] that exhibit therapeutic potential in preclinical research and ongoing clinical trials.

#### PD

The pathological features of PD are the loss of dopaminergic neurons in the substantia nigra and formation of Lewy bodies. Current treatments for PD mainly rely on drug therapy and auxiliary surgical treatment, which exhibits symptomatic attenuation in motor fluctuation [59]. The exploration of other therapeutic approaches for PD have shown promising results, such as stem cell therapy, immune and inflammatory therapy using antibodies, and gene therapy [60]. Stem cell transplantation prevents the loss of dopaminergic neurons the substantia nigra of PD patients. The main transplantation method is injecting the cells directly to the brain parenchyma via stereotactic injection, which is invasive. Thus, noninvasive INA has attracted much attention. Table 1 lists the research of intranasal stem cell treatment of PD in rodent models in recent years. In 2011, Danielyan et al. first attempted to treat PD with intranasal MSCs in a rat model with unilateral substantia nigra damage induced by 6-OHDA [56]. The MSCs entered, survived, and proliferated in brain parenchyma for at least 4.5 months. Intranasal transplantation of MSCs effectively improved the tyrosine hydroxylase (TH) level in the damaged substantia nigra and striatum, and the motor symptoms in the model rats [56]. Subsequently, BM-MSCs, NSCs, DPSCs, and endometrial MSCs were administered intranasally to treat PD model rodents. And all of them produced some therapeutic effects [46, 49, 50, 62, 63]. However, the entry pathway, influencing factors and the different effect of different type of intranasally injected stem cells, have not been clarified and are worthy of further exploration.

**Table 1:** Recent studies on the treatment of PD model by INA of stem cells

Authors	Year	Animal models	Cell types	No. of cells per animal	Markers	Results
Danielyan et al. [56]	2011	Unilateral 6-OHDA rat model	MSCs	1 × 10^6^	EGFP	MSCs survived for at least 4.5 months and began to proliferate. TH levels in damaged-side substantia nigra and motor symptoms were improved.
Bossolasco et al. [61]	2012	Bilateral 6-OHDA rat model	hMSCs	2 × 10^6^	NIR815	Strong near infrared signals observed in the olfactory epithelium 10 min after INA, which moved inside the nose within 30 min and disappeared after 1 h. No signal in coronal section of the brain.
Danielyan et al. [62]	2014	(Thy1)-h [A30P] αS mice	BM-MSCs	1 × 10^6^	EGFP	EGFP-expressing MSCs detected in olfactory bulb, cortex, amygdala, striatum, hippocampus, cerebellum, and brainstem 7 days after INA. Predominant distribution in olfactory bulb and brainstem.
Salama et al. [63]	2017	Rotenone mouse model	MSCs	5 × 10^5^	None	At 70 days after INA, striatum TH staining showed the density of TH fibers was protected by MSCs, proving their protective effect on dopaminergic neurons.
Li et al. [46]	2017	MPTP mouse model	BM-NSCs	3 × 10^5^	None	At 14–15 days after INA, animals’ behavior improved significantly, and combination therapy with fasudil enhanced this effect.
Bagheri-Mohammadi et al. [50]	2019	Bilateral 6-OHDA mouse model	hEDSCs	1 × 10^5^, 5 × 10^5^, 1 × 10^6^	GFP	At 30, 60, 90, and 120 days after INA, animals’ behavior improved significantly, which was most significant in 5 × 10^5^ cells per animal group. The results of TH staining in striatum were improved significantly
Simon et al. [49]	2019	MPTP mouse model	DPSCs	5 × 10^5^	PKH26	At 7 days after INA, labeled cells were observed in the substantia nigra. By week 4, the TH staining of substantia nigra improved significantly. Obvious effect in several behavioral results were shown.

*BM-MSCs* bone marrow-derived mesenchymal stem cells, *BM-NSCs* bone marrow-derived neural stem cells, *hEDSCs* human endometrial mesenchymal stem cells.N

#### AD

AD is the most common neurodegenerative disease, which is pathologically characterized by accumulation of amyloid plaques formed by amyloid-β (Aβ) and neurofibrillary tangles consisting of hyperphosphorylated tau. Impaired synaptic plasticity and neurodegeneration have been observed in the brains of AD patients. Exogenous stem cells play an important role in the reconstruction and regeneration of failed neuronal circuits [64]. In 2014, the research group of Danielyan L utilized intranasal injection of BM-MSCs to treat AD transgenic APP/PS1 mice. On 7 days after INA, stem cells were found near the Aβ deposition in the hippocampus and occipital cortex. The transplanted cells were also Aβ positive, suggesting that they had phagocytic and scavenging effects on Aβ [62]. In 2015, Mita et al. found that after intranasal injection of the serum-free conditioned medium of stem cells derived from human exfoliated deciduous teeth (SHED-CM), the cognitive function of AD model mice was improved [65]. Intranasal injection of the soluble molecules [66] or exosomes [67] of MSCs ameliorates the inflammation of AD mice and reduces Aβ. Table 2 summarizes recent research of INA of stem cells in the treatment of AD model mice. Intranasal injection of MSCs improves the abnormal dopamine system and inflammatory response in Huntington’s disease [68].

**Table 2:** Recent studies on treatment of AD murine models by INA of stem cells

Authors	Year	Animal models	Cell types	Results
Danielyan et al. [62]	2014	APP/PS1 mouse model	BM-MSC-derived macrophages	At 7 days after INA, macrophages induced by BM-MSCs were detected in different brain regions affected by Aβ plaque pathology
Mita et al. [65]	2015	Aβ1–40-peptide induced mouse model	SHED-CM	Cognitive function effectively improved. Reduced proinflammatory response of Aβ. Created anti-inflammatory/tissue regeneration environment. Induced formation of the anti-inflammatory M2-like microglia.
Harach et al. [66]	2016	APP/PS1 mouse model	Soluble molecules of hMSC	Ten weeks INA, the level of Aβ was effectively reduced. Continuous intranasal injection of soluble factor of hMSC also reduced amyloidosis in mice brain.
Perets et al. [67]	2019	5xFAD transgenic mouse model	Exosomes of MSCs	Exosomes were observed to accumulate near Aβ plaques within 96 h after INA.

*SHED-CM* stem cells from human exfoliated deciduous teeth-conditioned medium

### Cerebrovascular diseases

Stem cell therapy also has advantages in the prognosis and functional repair of cerebrovascular diseases, especially in some ischemic vascular diseases. In vivo, peripheral vascular endothelial progenitor cells can repair damaged vascular endothelial cells and promote vascular regeneration [69]. In 2012, Wei et al. investigated intranasal transplantation of BM-MSCs in a murine model of ischemic stroke. The stem cells reached the ischemic cortex and were deposited outside the blood vessels at 1.5 h after administration. The transplanted stem cells homed to the infarcted area, protecting the cells in it [16]. The conditioned medium of nasal MSCs also improves the symptoms of middle cerebral artery occlusion MCAO model rats and enhance vascular remodeling [70]. INA of stem cells also exerts therapeutic effects in model rodents with subarachnoid hemorrhage [71] or neonatal brain injury [72–74].

### GBM

GBM is one of the most aggressive malignant tumors and has poor prognosis. Stereotactic injection of stem cells has produced encouraging results in the treatment of GBM. The poor repeatability and invasiveness of drug delivery limits its application. INA provides a good method of stem cell administration [15]. In 2012, Reitz et al. found that NSCs were detected around GBM tissues 6 h after INA, which reached a peak at 24 h and persisted in the brain for more than 5 days [44]. In 2016, Dey et al. significantly enhanced the ability of NSCs to target tumors by hypoxic preconditioning or overexpression of chemokine CXC receptor 4. The modified NSCs more effectively delivered oncolytic viruses to glioma and prolonged the survival time of experimental animals, in combination with radiotherapy [43]. Current studies suggest that intranasal stem cell therapy (especially NSCs), after combination with other treatments, has a good effect on the treatment and prognosis of GBM and has great prospects.

## Limitations of intranasal stem cell therapy

### Cell migration in the brain after INA

Fluorescent tracer technology can be used to monitor the distribution of cells after they enter the brain. As the first pioneers, Danielyan et al. intranasally delivered fluorescently labeled rat MSCs or human glioma cells to naïve mice and rats. The fluorescently labeled cells were found in the olfactory bulb, cerebellum, subarachnoid space, and cortex [56]. In another study by the same group, MSCs were distributed near Aβ in AD model mice [62]. Reitz et al. intranasally injected enhanced green fluorescent protein (eGFP)-labeled NSCs into a glioma mouse model and found the cells mainly accumulated near the tumor [44]. In our study to compare the biodistribution of human neural stem cell (hNSC) migration into the brain via INA, we found that hNSCs delivered by INA could migrate into the striatum, hippocampus, cerebellum, and spinal cord in MPTP (1-methyl-4-phenyl-1,2,3,6-tetrahydropyridine) treated mice, but they could not migrate into CNS after INA in normal mice [75]. An increasing number of studies have suggested that biodistribution differs among brain regions over time due differences in disease models and delivery and quantity of stem cells. There is no clear answer as to which method is best to label or trace stem cells. Some scholars believe that in vivo neuroimaging may be good [19, 76]. However, from the results of our previous studies [75], in vivo tracing by positron emission tomography/computed tomography is not satisfactory, and more study is needed. A few tracking studies have shown the enter path of stem cells. For instance, Galeano et al. have labeled hMSCs with bright quantum dots in order to track the cells efficiently [14]. The use of stem cell exosomes in nasal drops to treat neurological disease has also received considerable attention. In 2019, Perets et al. confirmed that the exosomes of MSCs administered intranasally selectively target brain tissues in different models of neurological diseases [67]. This indicates that some secretory substances play an important role in the migration and homing of intranasally injected stem cells.

### Efficiency and quantity of stem cell entry

Not all stem cells reach the brain parenchyma after INA. Carlos et al. showed that among the intranasally injected MSCs, only a small number entered the CNS through the cribriform plate, while most fell into the gap of the turbinate bones [14]. Danielyan et al. have used quantitative polymerase chain reaction analysis of eGFP DNA to show the yield of eGFP-MSCs after INA [56, 62]. They used 1*10^6^ MSCs by INA, but only 1*10^4^ were found in the olfactory bulb, 100 in the hippocampus and 10 in the cortex. Therefore, further research is needed to improve the efficiency of cell delivery. Some experiments have shown that hyaluronidase can greatly the permeability of olfactory epithelium and improve material utilization rate. This method has also been used in intranasal stem therapy [12, 15]. It should be noted that different concentrations of stem cells were used across the studies. Thus, does the initial cell concentration affect experimental results? A study on PD reported that the behavioral improvement in the low stem cell concentration treatment group was better than that of the high concentration group [50], but this result needs validation.

### Body weight and age of model animals

The distribution of drugs or cells after intranasal injection exhibits variability in distinct animal models. Even in the same animal model, intranasal injection shows different delivery efficacy according to animal age and weight. Krishnan et al. injected the drug radiolabeled pralidoxime into the nasal cavity of model animals, which showed that compared with younger animals with lower body weight, older animals with higher body weight required a higher dose to reach the same drug concentration in the whole brain [77]. This suggested that the distribution of intranasally administered stem cells might be affected by the age and body weight of model animals.

## Conclusions: summary and prospects

INA has the advantages of noninvasiveness and high practicability. It facilitates all kinds of drug molecules and stem cells to bypass the BBB and reach the CNS, which provides a noninvasive and effective method for stem cell therapy in CNS diseases. However, unlike small molecular substances, the transnasal transport routes of stem cells remain unclear. Optimization of approaches in improving the delivery and therapeutic efficiency and safety of intranasal transplantation of stem cells is required in future studies. There are still many issues to be considered in the translation from animal experiments to clinical trials (Table 3).

**Table 3:** Challenges of stem cells translation from animal experiments to clinical application via INA

Challenges	Solutions
Long-term therapeutic effect of cell transplantation	Long-term observations of therapeutic effect in different animal models
Optimum cell quantity	Different optimal cell sizes need to be found under different models
Long-term safety of cell transplantation	Long-term observations of side effect in different animal models (such us oncogenicity)
Appropriate disease type	Explore more possible treatments for neurological diseases

## Data Availability

Data sharing is not applicable to this article as no datasets were generated or analyzed during the current study.

## Abbreviations

Aβ: Amyloid-β
AD: Alzheimer’s disease
BBB: Blood–brain barrier
BM-MSCs: Bone-marrow-derived MSCs
CNS: Central nervous system
DPSCs: Dental pulp stem cells
hNSCs: Human neural stem cells
INA: Intranasal application
iPSCs: Induced pluripotent stem cells
MSCs: Mesenchymal stem cells
NSCs: Neural stem cells
ORNs: Olfactory receptor neurons
PD: Parkinson’s disease
SVZ: Subventricular zone
TH: Tyrosine hydroxylase
vWF: von Willebrand factor
WGA-HRP: Wheat Germ Agglutinin
